# Patterns and Trends of Glucose‐Lowering Therapy in Alzheimer's Disease and Related Dementias

**DOI:** 10.1111/dom.70817

**Published:** 2026-05-12

**Authors:** Huilin Tang, Bingyu Zhang, Yiwen Lu, Ying Lu, Anastassia Amaro, Yuan Lu, David A. Wolk, Yong Chen

**Affiliations:** ^1^ The Center for Health AI and Synthesis of Evidence (CHASE), University of Pennsylvania Philadelphia Pennsylvania USA; ^2^ Department of Biostatistics, Epidemiology, and Informatics University of Pennsylvania Perelman School of Medicine Philadelphia Pennsylvania USA; ^3^ The Graduate Group in Applied Mathematics and Computational Science, School of Arts and Sciences University of Pennsylvania Philadelphia Pennsylvania USA; ^4^ Penn Metabolic Medicine, Division of Endocrinology University of Pennsylvania Philadelphia Pennsylvania USA; ^5^ Section of Cardiovascular Medicine, Department of Medicine Yale School of Medicine New Haven Connecticut USA; ^6^ Center for Outcomes Research and Evaluation, Yale New Haven Health New Haven Connecticut USA; ^7^ Department of Neurology University of Pennsylvania Perelman School of Medicine Philadelphia Pennsylvania USA; ^8^ Leonard Davis Institute of Health Economics, University of Pennsylvania Philadelphia Pennsylvania USA; ^9^ Penn Medicine Center for Evidence Based Practice (CEP), university of Pennsylvania Philadelphia Pennsylvania USA; ^10^ Penn Institute for Biomedical Informatics (IBI), university of Pennsylvania Philadelphia Pennsylvania USA

**Keywords:** antidiabetic drug, cohort study, database research, elderly, glycaemic control

## 1 Background

Type 2 diabetes (T2D) frequently coexists with Alzheimer's disease and related dementias (ADRD) and complicates clinical management due to cognitive decline, polypharmacy and heightened risks of hypoglycemia and adverse events [1, 2]. Optimizing glycemic management in this population requires balancing efficacy and safety while accounting for heterogeneity in functional status and life expectancy. Newer glucose‐lowering drugs (GLDs), including glucagon‐like peptide‐1 receptor agonists (GLP‐1RAs) and sodium–glucose cotransporter‐2 inhibitors (SGLT2is), have demonstrated cardiovascular and renal benefits, with potential neuroprotective effects [3]. However, their uptake and use in adults with ADRD remain poorly characterized in real‐world settings, where concerns about tolerability, administration burden and clinical complexity may influence prescribing. Using a large, real‐world, multicenter electronic health record network spanning more than a decade, we evaluated national trends in the use of traditional and newer GLDs among adults with T2D and ADRD from 2014 to 2025.

## 2 Methods

We conducted a retrospective cohort study using individual‐level, de‐identified electronic health record (EHR) data from TriNetX research network. TriNetX is a global health research platform that aggregates de‐identified EHR data from multiple healthcare organizations, primarily across the United States, including hospitals, primary care and specialty clinics [4]. The database captures longitudinal patient information, such as demographics, diagnoses, procedures, laboratory results, medications and clinical outcomes, enabling comprehensive real‐world analyses. The University of Pennsylvania Institutional Review Board deemed this cohort study exempt from review and informed consent.

Adults aged ≥ 50 years with diagnoses of both T2D (ICD‐10‐CM: E11) and ADRD (ICD‐10‐CM: F01, F02, F03, F09, G31.0, G31.1, G31.83), between January 1st, 2014, and July 31st, 2025, were included. The index date was defined as the first date when patient had documented diagnoses of both T2D and ADRD. Patients were followed from the index date until death, last encounter, or 1 year, whichever occurred first, to identify the first prescription of any GLD use after cohort entry, irrespective of prior treatment history. GLDs were categorized as insulin, metformin, GLP‐1RAs, SGLT2is, sulfonylureas, dipeptidyl Peptidase‐4 Inhibitors (DPP4is), other agents (e.g., alpha‐glucosidase inhibitors, meglitinides), or combination (e.g., 2 or ≥ 3 GLDs).

At the patient level, we calculated prescription proportions and annual trends in GLD use. Baseline characteristics, assessed within 1 year prior to the index date, including demographics, comorbidities, prior GLD use and clinical measures (body mass index [BMI] and HbA1c), were summarized using descriptive statistics and compared between groups using standard mean difference with value < 0.1 indicating balance. Temporal trends in GLD use were assessed using the Cochran‐Armitage test and visualized over the follow‐up period. All analyses were conducted using R version 4.5.0.

## 3 Results

**FIGURE 1:**
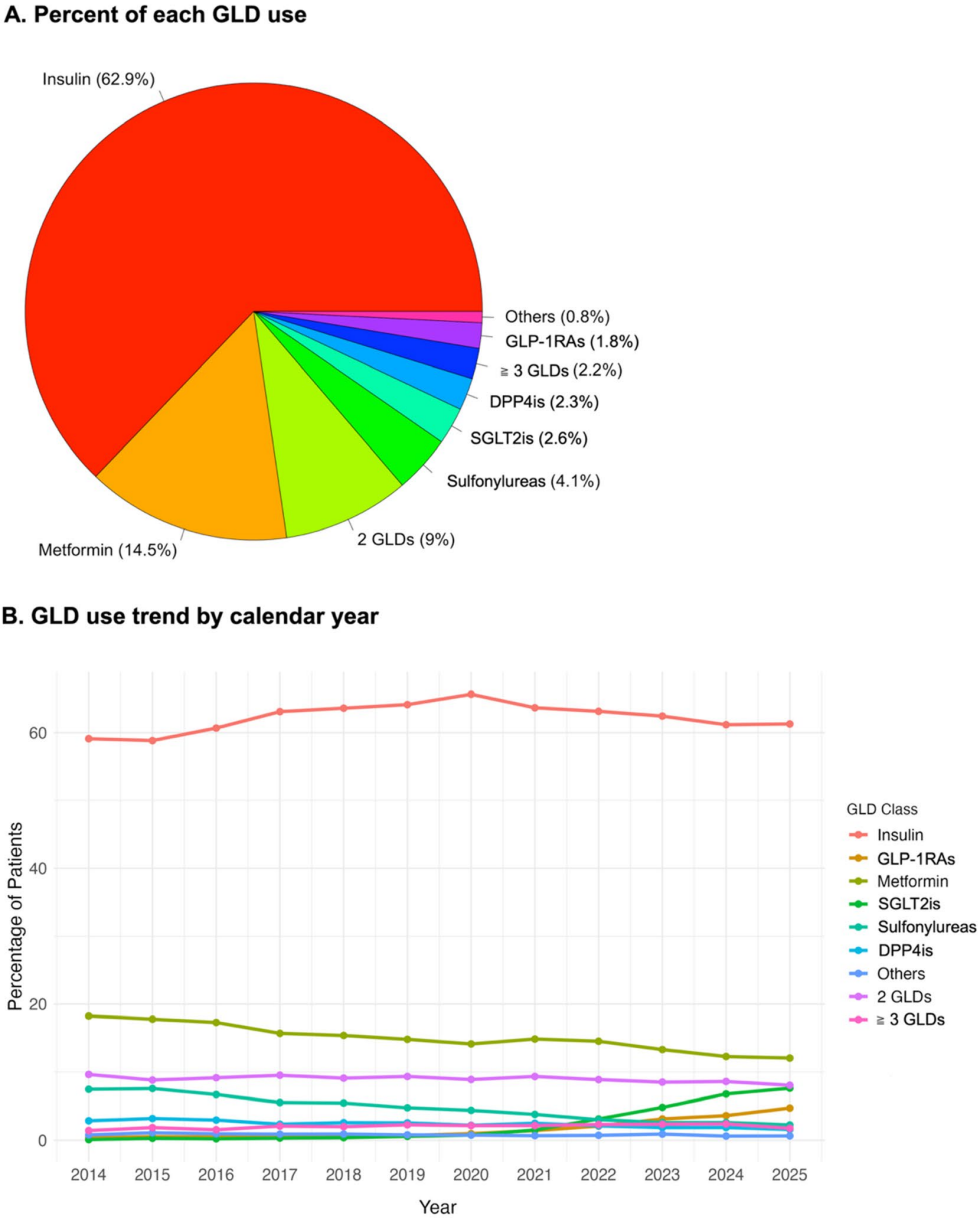
Patient‐level patterns and temporal trends of glucose‐lowering drug use among patients with type 2 diabetes and Alzheimer's Disease‐Related Dementias, 2014–2025. Panel A shows the proportions of patients receiving prescriptions of each GLD class within 1 year of diagnosis; Panel B displays the percentage of patients with GLD prescriptions from 2014 to 2025. GLD, glucose‐lowering drug; GLP‐1RAs, glucagon‐like peptide‐1 receptor agonists; SGLT2is, sodium–glucose cotransporter‐2 inhibitors; DPP4is, dipeptidyl peptidase‐4 inhibitors; Others, including alpha‐glucosidase inhibitors and meglitinides.

Among 387 042 adults with T2D and ADRD (mean [SD] age, 75.5 [8.2] years; 46.0% men; 43.7% non‐Hispanic White), only 185 093 (47.8%) received a GLD prescription within 1 year of the index date (Table 1). Among GLD users, insulin was the most frequently prescribed (62.9%), followed by metformin (14.5%), dual‐agent combinations (9.0%), sulfonylureas (4.1%), SGLT2is (2.6%) and GLP‐1RAs (1.8%) (Figure 1A). The baseline characteristics differed substantially across groups (Table 1). Patients prescribed GLP‐1RAs were generally younger and had higher BMI. Comorbidities were highly prevalent overall, particularly hypertension, hyperlipidemia and ischemic heart disease. Heart failure and chronic kidney disease were particularly prevalent among insulin and SGLT2i users. Patients receiving combination therapy had a higher baseline HbA1c level (7.7%–8.0%).

**TABLE 1:** Demographic characteristics of patients with type 2 diabetes and Alzheimer's disease and related dementias by glucose‐lowering drug use.

	No use	Insulin	Metformin	GLP‐1RAs	SGLT2is	DPP4is	SUs	Other GLDs	2 GLDs	≥ 3 GLDs	SMD*
*n*	201 949	116 337	26 748	3338	4853	4214	7548	1474	16 574	4007	
Age, years, mean (sd)	75.7 (8.2)	75.4 (8.1)	74.5 (8.2)	71.2 (8.9)	75.7 (8.3)	76.5 (7.6)	76.4 (7.5)	76.3 (7.8)	75.2 (8.0)	75.0 (8.1)	0.19
Age ≥ 65 years	180 586 (89.4)	103 782 (89.2)	23 248 (86.9)	2575 (77.1)	4343 (89.5)	3868 (91.8)	6971 (92.4)	1348 (91.5)	14 804 (89.3)	3554 (88.7)	0.123
Male	90 158 (46.5)	55 145 (49.3)	12 403 (47.5)	1413 (43.9)	2638 (55.8)	1744 (42.8)	3609 (49.0)	677 (47.7)	8167 (50.5)	2076 (52.9)	0.092
Race/ethnicity											
Hispanic	15 541 (7.7)	8196 (7.0)	1702 (6.4)	236 (7.1)	371 (7.6)	344 (8.2)	365 (4.8)	115 (7.8)	1188 (7.2)	323 (8.1)	0.151
NHW	86 318 (42.7)	52 980 (45.5)	11 875 (44.4)	1719 (51.5)	2233 (46.0)	1733 (41.1)	3280 (43.5)	670 (45.5)	6988 (42.2)	1513 (37.8)	
NHB	30 404 (15.1)	17 732 (15.2)	3023 (11.3)	345 (10.3)	668 (13.8)	600 (14.2)	821 (10.9)	160 (10.9)	2000 (12.1)	350 (8.7)	
Others	69 686 (34.5)	37 429 (32.2)	10 148 (37.9)	1038 (31.1)	1581 (32.6)	1537 (36.5)	3082 (40.8)	529 (35.9)	6398 (38.6)	1821 (45.4)	
Region											
Midwest	37 981 (18.8)	21 318 (18.3)	5445 (20.4)	650 (19.5)	947 (19.5)	612 (14.5)	1949 (25.8)	304 (20.6)	2910 (17.6)	593 (14.8)	0.289
Northeast	51 274 (25.4)	26 602 (22.9)	6284 (23.5)	872 (26.1)	1202 (24.8)	1267 (30.1)	1678 (22.2)	419 (28.4)	3915 (23.6)	970 (24.2)	
South	73 830 (36.6)	45 288 (38.9)	8152 (30.5)	1139 (34.1)	1692 (34.9)	1346 (31.9)	2306 (30.6)	465 (31.5)	5266 (31.8)	1075 (26.8)	
Unknown	13 827 (6.8)	8177 (7.0)	2436 (9.1)	47 (1.4)	211 (4.3)	282 (6.7)	763 (10.1)	49 (3.3)	2338 (14.1)	823 (20.5)	
West	25 037 (12.4)	14 952 (12.9)	4431 (16.6)	630 (18.9)	801 (16.5)	707 (16.8)	852 (11.3)	237 (16.1)	2145 (12.9)	546 (13.6)	
Comorbidities at baseline											
T2D duration, years, mean (sd)	2.1 (3.3)	2.9 (4.1)	2.7 (3.9)	4.6 (4.8)	3.7 (4.4)	3.2 (4.2)	3.1 (4.0)	3.3 (4.2)	2.6 (4.0)	2.5 (4.0)	0.191
ADRD duration, years, mean (sd)	0.3 (1.2)	0.1 (0.8)	0.3 (1.3)	0.4 (1.7)	0.4 (1.5)	0.1 (0.7)	0.1 (0.7)	0.1 (1.1)	0.1 (1.0)	0.1 (0.6)	0.131
IHD	68 225 (33.8)	55 219 (47.5)	7236 (27.1)	1048 (31.4)	2554 (52.6)	1347 (32.0)	2506 (33.2)	414 (28.1)	6349 (38.3)	1463 (36.5)	0.193
PVD	37 067 (18.4)	26 775 (23.0)	3523 (13.2)	635 (19.0)	1426 (29.4)	706 (16.8)	1305 (17.3)	224 (15.2)	2701 (16.3)	552 (13.8)	0.133
Heart failure	47 646 (23.6)	42 238 (36.3)	3819 (14.3)	654 (19.6)	2460 (50.7)	907 (21.5)	1638 (21.7)	231 (15.7)	4300 (25.9)	929 (23.2)	0.262
Cerebrovascular disease	52 031 (25.8)	37 937 (32.6)	5868 (21.9)	782 (23.4)	1418 (29.2)	1010 (24.0)	1727 (22.9)	321 (21.8)	4437 (26.8)	1046 (26.1)	0.09
Hypertension	162 520 (80.5)	103 477 (88.9)	21 730 (81.2)	2780 (83.3)	4294 (88.5)	3544 (84.1)	6438 (85.3)	1217 (82.6)	14 397 (86.9)	3504 (87.4)	0.102
Hyperlipidemia	123 827 (61.3)	81 057 (69.7)	18 281 (68.3)	2502 (75.0)	3827 (78.9)	2954 (70.1)	5231 (69.3)	1055 (71.6)	11 651 (70.3)	2886 (72.0)	0.11
CKD	59 560 (29.5)	51 241 (44.0)	4391 (16.4)	1022 (30.6)	2111 (43.5)	1538 (36.5)	2664 (35.3)	508 (34.5)	5127 (30.9)	1098 (27.4)	0.202
Overweight/Obesity	32 542 (16.1)	26 705 (23.0)	4631 (17.3)	1332 (39.9)	1292 (26.6)	683 (16.2)	1328 (17.6)	246 (16.7)	3454 (20.8)	823 (20.5)	0.174
Cancer	26 811 (13.3)	15 095 (13.0)	2951 (11.0)	434 (13.0)	716 (14.8)	525 (12.5)	915 (12.1)	175 (11.9)	1698 (10.2)	362 (9.0)	0.059
GLD use at baseline											
Insulin	18 099 (17.9)	46 471 (60.2)	3937 (19.2)	1033 (34.3)	1104 (27.0)	890 (27.3)	1318 (22.7)	305 (25.8)	4974 (46.9)	1211 (50.9)	0.366
Metformin	9283 (9.2)	13 044 (16.9)	13 251 (64.6)	925 (30.7)	898 (22.0)	781 (24.0)	1515 (26.1)	306 (25.9)	4584 (43.2)	1291 (54.2)	0.453
GLP‐1RAs	1403 (1.4)	3013 (3.9)	678 (3.3)	1846 (61.3)	363 (8.9)	87 (2.7)	213 (3.7)	82 (6.9)	928 (8.7)	371 (15.6)	0.452
SGLT2is	2082 (2.1)	3462 (4.5)	627 (3.1)	477 (15.8)	2111 (51.6)	177 (5.4)	270 (4.7)	79 (6.7)	1274 (12.0)	495 (20.8)	0.422
DPP4is	1993 (2.0)	4115 (5.3)	755 (3.7)	161 (5.3)	245 (6.0)	1846 (56.6)	409 (7.1)	124 (10.5)	1507 (14.2)	609 (25.6)	0.448
SUs	3454 (3.4)	6377 (8.3)	1651 (8.1)	335 (11.1)	383 (9.4)	508 (15.6)	3728 (64.3)	212 (17.9)	2748 (25.9)	966 (40.6)	0.519
Other GLDs	778 (0.8)	1517 (2.0)	341 (1.7)	114 (3.8)	113 (2.8)	122 (3.7)	196 (3.4)	706 (59.7)	663 (6.2)	309 (13.0)	0.438
Other values (latest value at baseline)											
BMI, mean (sd)	27.5 (5.6)	27.6 (5.7)	28.0 (5.5)	31.3 (5.6)	28.1 (5.5)	27.7 (5.4)	28.2 (5.6)	28.0 (5.6)	27.7 (5.7)	27.7 (5.8)	0.167
Hba1c, mean (sd)	6.5 (1.3)	7.4 (1.6)	6.9 (1.2)	7.3 (1.5)	7.0 (1.4)	7.2 (1.3)	7.3 (1.4)	7.3 (1.4)	7.7 (1.6)	8.0 (1.6)	0.323

*Note:* Values are numbers (percentages) unless otherwise indicated.

Abbreviations: ADRD, Alzheimer's disease and related dementias; BMI, body mass index; CKD, chronic kidney disease; DPP4is, Dipeptidyl Peptidase‐4 Inhibitors; GLD, glucose‐lowering drugs; GLP‐1RAs, glucagon‐like peptide‐1 receptor agonists; IHD, Ischemic heart disease; NHB, Non‐Hispanic Black; NHW, Non‐Hispanic Whites; Other GLDs, including alpha‐glucosidase inhibitors and meglitinides; PVD, Peripheral vascular disease; SGLT2is, sodium–glucose cotransporter‐2 inhibitors; SUs, sulfonylureas; T2D, type 2 diabetes.

* SMD, standard mean difference, with value < 0.1 indicating balance.

Temporal trends in patient‐level prescribing patterns from 2014 to 2025 showed gradual uptake of newer agents (Figure **1**B). SGLT2i use increased markedly from 0.1% to 8.0% with an approximately 80‐fold rise (*p* < 0.001), while GLP‐1RA prescription increased from 0.5% to 5.0% (approximately 10‐fold increase; *p* < 0.001). In contrast, insulin use peaked in 2020 at nearly 70% of all GLD prescriptions before showing a slight decline thereafter, with no significant temporal trend over the study period (*p* = 0.33). Use of metformin (*p* < 0.001), sulfonylureas (*p* < 0.001) and DPP4is (*p* < 0.001) showed statistically significant but modest declining trends. The proportion of patients receiving combination therapy remained stable at approximately 10%.

## 4 Discussions

In this large real‐world cohort of adults with T2D and ADRD, we observed a substantial shift in GLD prescribing patterns, characterized by rapid uptake of newer agents and gradual decline of several older therapies. Specifically, SGLT2is and GLP‐1RAs showed marked and sustained increases in use, whereas sulfonylureas, DPP4is, and metformin showed modest but statistically significant declines. Insulin use remained relatively stable over time. These findings reflect a broad evolution in diabetes pharmacotherapy over the past decade.

The rapid adoption of SGLT2is and GLP‐1RAs likely reflects evidence from cardiovascular and renal outcome trials and guideline recommendations favouring their use in patients with T2D, particularly those with cardiorenal disease [2, 5]. Beyond metabolic benefits, emerging evidence suggests potential neuroprotective effects of both drug classes [3], with observational studies suggesting possible associations with reduced cognitive decline, although causal evidence remains limited [6]. However, despite these potential benefits, uptake of these newer agents remained relatively limited, suggesting delayed translation of evidence into routine clinical practice. This gap may be driven by cost barriers, limited familiarity, tolerability concerns and uncertainty regarding long‐term benefit–risk profiles in older adults with ADRD [7].

In contrast, the declining use of sulfonylureas and DPP4is is consistent with their comparatively limited cardiometabolic benefits and, for sulfonylureas, higher risk of hypoglycemia [8], a particularly important consideration in patients with ADRD [9]. The modest decline in metformin use may reflect earlier initiation of combination therapy and the greater clinical complexity of this population. Insulin use remained stable over time, consistent with its continued role in advanced disease and glycemic management when oral and injectable non‐insulin agents are insufficient [5]. Overall, combination therapy use also remained stable at approximately 10%, suggesting limited change in treatment intensification patterns.

This study has several strengths, including a large cohort and an extended 11‐year observation period. However, limitations should be acknowledged. Misclassification of diagnoses and prescriptions is possible, and information on medication dosing and frailty was not available. In addition, this study was descriptive in nature and aimed to characterize prescribing patterns and temporal trends rather than to assess determinants of treatment choice; therefore, adjusted analyses and sensitivity analyses were not performed, and findings should not be interpreted causally. Future studies should examine patient‐ and clinician‐level factors driving the selection of specific GLD (e.g., GLP‐1RAs or SGLT2is) in this population to inform individualized treatment strategies.

In conclusion, this study show a clear transition towards newer cardioprotective and potentially neuroprotective GLDs in adults with T2D and ADRD, alongside persistent delays in their early adoption and limited treatment intensification in routine care. Targeted implementation strategies and pragmatic trials are needed to optimize diabetes care and reduce treatment‐related harms in this vulnerable population.

## Author Contributions

H.T. and Y.C. were involved in the study design. H.T. and Ying Lu conducted the statistical analysis. All authors participated in the interpretation of study results and in the critical revision and approval of the final version of the manuscript.

## Funding

This work was supported in part by the National Institutes of Health (F1AG077820, R01AG073435, R56AG074604).

## Conflicts of Interest

A.A. reported funding and consulting fees from Novo Nordisk, outside the submitted work. Y.L. reported receiving grants from NHLBI, PCORI, Novartis and Sentara Research Foundation outside the submitted work. D.W. reported grants from the National Institutes of Health during the conduct of the study and personal fees from Eli Lilly, Beckman Coulter, and GSK outside the submitted work. Y.C. reported grants from the National Institutes of Health during the conduct of the study and personal fees from Merck and Pfizer outside the submitted work. All other authors declare no conflicts of interest.

## Data Availability

The data could be obtained on a reasonable request. Further information about the data of TriNetX can be accessed on their website: https://trinetx.com/?mc_cid=7e2ecd5bc5&mc_eid=%5BUNIQID%5D.
